# Visual Maturation at Term Equivalent Age in Very Premature Infants According to Factors Influencing Its Development

**DOI:** 10.3389/fphys.2018.01649

**Published:** 2018-11-20

**Authors:** Maëlle Wirth, Aurélie Naud, Emmanuelle Schmitt, Isabelle Clerc-Urmès, Jean-Michel Hascoët

**Affiliations:** ^1^Department of Neonatology, Centre Hospitalier Régional Universitaire de Nancy, Nancy, France; ^2^EA 3450 DevAH, University of Lorraine, Nancy, France; ^3^Department of Neuroradiology, Centre Hospitalier Régional Universitaire de Nancy, Nancy, France; ^4^PARC Clinical Research Support Facility, Centre Hospitalier Régional Universitaire de Nancy, Nancy, France

**Keywords:** visual maturation, preterm infants, cerebral MRI, cerebral volume, retinal vascularization, retinopathy of prematurity, visual acuity

## Abstract

**Introduction:** Visual impairment is a concern in premature infants as perinatal factors may alter maturation during visual development. This observational study aimed at evaluating visual maturation at term equivalent age and factors associated with impaired visual maturation.

**Methods:** Infants born before 32 weeks’ gestation were evaluated with routine brain MRI, visual acuity, refraction, fundus, and clinical eye examination. Environmental factors were collected from infant’s files.

**Results:** Fifty-four infants (29.5 ± 1.7 weeks’ gestation, birth weight 1194 ± 288 g) were studied at term equivalent age. Visual acuity was higher in premature infants at term equivalent age than in a reference publication with the same method in term newborns at birth (1.54 ± 0.67 vs. 0.99 ± 0.40 cycles/degree, *p* = 0.008). In multivariate analysis, abnormal brain MRI was the only factor associated with visual acuity (*r*^2^= 0.203; *p* = 0.026). Incomplete retinal vascularization was observed in 29/53 of infants at term equivalent age and associated with MRI abnormalities of the posterior fossa (*p* = 0.027) and larger refractive sphere difference between both eyes (1.2 ± 0.8 vs. 0.6 ± 0.4 diopters; *p* = 0.0005). Retinopathy of prematurity was associated with indices of smaller cerebral volume (*p* = 0.035).

**Conclusion:** Higher visual acuity in premature infants at term equivalent age than in term newborns at birth may be related to longer visual experience from birth. Lower visual acuity was correlated with abnormal MRI in preterm infants at term equivalent age.

## Introduction

Visual impairment is observed in 3% of preterm infants born before 37 weeks GA. The main related causes are ROP, refractive ametropia, strabismus, and visual cortex involvement. The risk of visual impairment is higher when the prematurity is more important. Hellgren et al. (2016) showed 2.1% of blindness, 4.8% of visual impairment according to the World Health Organization in a cohort of infants born before 27 weeks GA at the 6.5-year follow-up. Moreover, they presented in comparison with the term control children group at age 6.5 years 17.4% vs. 0% of strabismus, 29.7% vs. 5.9% of refractive errors, 37.5% vs. 6.2% of ophthalmologic abnormalities (Hellgren et al., 2016).

Visual development occurs in three steps. The first step is the anatomical formation of the eye, which is influenced by genetics; the second step is the visual experience related to endogenous stimulation *in utero*; and the third step is the visual experience by exogenous stimulation after birth (Graven, 2004). Thus, VPI are at high risk of visual development vulnerabilities because the second step of development is curtailed and the third step begins in an immature system. Moreover, at 2 years of age, VPI born before 27 weeks GA with visual impairment have more psychomotor delay than those without visual impairment (Holmström et al., 2014). Therefore, visual maturation, defined as retinal, globe, vascular and non-vascular structures growth, and factors influencing this development are important in this population and have to be taken into consideration. However, the normal visual development in premature infants and the impact of the factors that influence it are poorly understood.

The aim of this study was to describe visual maturation of VPI at TEA and to investigate factors associated with impaired visual maturation.

## Materials and Methods

### Subjects

In our level III NICU, all preterm infants born before 32 weeks GA undergo routine MRI and an ophthalmological evaluation at TEA as part of their follow-up. Infants with a MRI scheduled between February and June 2016 were included in the present observational study. Infants with a genetic syndrome, ocular abnormality, facial malformation, or craniosynostosis were excluded.

### Primary Outcome

Primary outcome was the measurement of visual acuity at TEA. Given the early age of the infants, we measured discriminative visual acuity in cycles/degree using the preferential looking method and Baby-Vision^®^ acuity cards (Tropique, Paris, France) (Vital-Durand and Cottard, 1985). The infants sat on the lap of an adult as the examiner presented them with a series of cards. As appropriate for the age of the infants, the distance of the examination was 40 cm from the child. All examinations were carried out by the same investigator in the same quiet room with a bright and monotonous visual environment.

The examination was performed in binocular vision. The exam began with a low-frequency spatial acuity card and continued until the wandering glance. The answer was considered positive when the cards were perceived by the child. In response to a negative or doubtful answer, the examiner showed the previous cards again until the highest card was acknowledged. The visual acuity was that for which at least three of four responses were obtained.

### Secondary Outcomes

#### Visual Maturation Evaluation

Visual maturation evaluation included clinical eye examination evaluating the ocular anatomy (appearance of the eyeballs, pupillary reflections, corneal aspect, palpebral aspect, appearance of sclera, conjunctiva, and iris). Signs of visual deficit were sought. Ocular motricity was evaluated in binocular vision.

Different reflexes were evaluated, including attraction to light, direct and consensual photomotor reflex, reflex of closing the eyelids to glare, fixation reflex, reflex of blinking to the threat, eye pursuit, reflex of convergence. Refraction was measured by an autorefractometer (Retinomax 3^®^, Righton, United States) under cycloplegia using 0.5% tropicamide drops. A retinal camera (RetCam Shuttle^®^, Clarity Medical Systems, United States) was used for FE as part of a longitudinal evaluation in the context of the routine screening and follow-up of ROP. It allowed to evaluate the maturation of the retinal vascularization, specifically with the completeness or incompleteness of the vascularization up to the ora serrata, the presence of ROP, and papillary excavation.

#### Neuroradiology

We also evaluated cerebral MRI data at TEA. Prior to undergoing MRI, each infant was fed, wrapped in a blanket, and placed (not sedated) in a 1.5 Tesla Vantage Titan^®^ of Toshiba tunnel with cardiac frequency and oxygen saturation monitoring. MRI was categorized as “MRI globally normal” or “MRI globally abnormal” when lesions were present, such as supra-tentorial injury (sequelae of germinal matrix hemorrhage, subependymal hemorrhage, and diffuse or cystic periventricular leukomalacia) and sub-tentorial or fossa posterior lesions. The following indices of cerebral volume were measured: CSI and VSA (Naud et al., 2017).

### Clinical and Pharmacological Data

Data related to the perinatal period were collected from the infants’ medical files in a standardized manner. The characteristics of the infants (GA, gender, Apgar score, weight and head circumference at birth and TEA), the therapies used and the major complications of prematurity PDA, intraventricular hemorrhage diagnosed by intracranial ultrasound and defined according to Papile’s classification (Papile et al., 1978), cystic periventricular leukomalacia diagnosed by MRI, BPD defined by Jobe and Bancalari (2001), ROP according to the international classification (International Committee for the Classification of Retinopathy of Prematurity, 2005), necrotizing enterocolitis defined by Bell (1978), early onset sepsis defined by sepsis ≤ 3 days of birth and late onset sepsis defined by sepsis > 3 days of birth, blood inflammation defined by C-reactive protein > 30 mg/l or Interleukin-6 > 250 μg/l, nutrition during the infants hospitalization classified by breastfeeding, artificial milk or mixed feeding, and the duration of neonatal hospitalization were recorded.

### Statistical Analysis

Visual acuity and secondary outcomes were described first. Data are presented as mean ± SD, or median and interquartile range and range. Next, visual acuity was compared to published reference data for children born at term (Vital-Durand, 1992). In order to compare the individual relationships between visual maturation and the perinatal environment, parametric (Student’s T, ANOVA, chi-square) and non-parametric tests (Mann and Whitney, Kruskal–Wallis, Fisher exact) were used when appropriate. To determine which variables were associated with visual acuity, bivariate and multivariate linear regression analyses were performed. Only the factor presenting a significant association at the threshold of 0.2 in the bivariate model were included in the multivariate model with the stepwise method (SLE = 0.2; SLS = 0.05). The threshold for significance was set at 5%. These analyses were performed by the clinical research support facility of the Nancy CHRU (Unit of Methodology, Data Management, and Statistics) using SAS v9.4 software (SAS Institute, Cary, NC, United States).

### Ethics Statement

This prospective observational study was approved by our institutional review board (DRI, CHRU Nancy: PSS2016/PREMAVISION) and registered with the Commission Nationale Informatique et Libertés (number: R2016-02) and ClinicalTrials.gov (number: NCT02890251). Both parents were informed that the data will be used for research purposes and their consent obtained in writing and signed. The research followed the tenets of the Declaration of Helsinki.

## Results

### Population Characteristics

Fifty-four infants were studied. The characteristics of our population in the neonatal period and at TEA evaluation are presented in Tables 1, 2, respectively. The duration of neonatal hospitalization was 68.5 ± 21.8 days.

**Table 1:** Neonatal characteristics of the study population (*N* = 54).

Variable	*N*	% or mean ±*SD*
Gestational age (weeks GA)	54	29.5 ± 1.7
Birthweight (grams)	54	1194 ± 288
Head circumference at birth (cm)	54	26.4 ± 1.9
Male	26	48.1
Apgar score at 1 min after birth	54	4.8 ± 2.5
Apgar score at 5 min after birth	54	6.8 ± 1.5
Antenatal corticosteroid	50/54	92.6
Intraventricular hemorrhage	8	14.8
Periventricular leukomalacia	0	0
Mechanical ventilation	50	92.6
Bronchopulmonary dysplasia	28	51.9
Mild	12	42.9
Moderate	13	46.4
Severe	3	10.7
Surgery for patent ductus arteriosus	5	9.3
Early onset infection	4	7.4
Late onset infection	16	29.6
Blood inflammation	16	29.6
Jaundice	45	83.3
Necrotizing enterocolitis, all stages	8/53	15.1
ROP, all stages	13/52	25.0
Maternal smoking during pregnancy	16/53	30.2
Maternal consumption of alcohol during pregnancy	2/53	3.8
Exclusive breastfeeding	18	33.3
Exclusive artificial feeding	1	1.9
Mixed feeding	35	64.8


**Table 2:** Characteristics of the study population at evaluation.

	*N*	%	Mean	*SD*	Median	Q1	Q3	Minimum	Maximum
Age (days)	54		90	15	89	81	95	64	133
Corrected age (weeks GA)	54		42.3	1.6	42.4	41.3	43.3	38.9	47.6
Weight (g)	54		3381	535	3370	2980	3780	2420	4400
Head circumference (cm)	54		35.8	1.4	36.0	35.0	36.8	32.0	38.5
Ophthalmologic family history	37/52	71.2							
Eyelid anomalies	7/54	13							
Cornea anomalies	0/54	0							
Sclera anomalies	0/54	0							
Conjunctiva anomalies	0/54	0							
Iris anomalies	0/54	0							
Eyeballs anomalies	0/54	0							
Pupillary reflections anomalies	0/54	0							
Signs of visual deficit	0/54	0							
Eye pursuit anomalies	0/54	0							
Attraction to light	54/54	100							
Closure of the eyelids to glare	53/54	98.1							
Reflex of blinking to the threat	49/53	92.5							
Reflex of convergence	8/53	15.1							
Reflex of fixation	54/54	100							
Binocular visual acuity (cycles/degree)	52		1.5	0.7	1.6	0.8	1.9	0.5	2.4
Refraction, sphere, right eye (D)	54		3.5	1.7	3.4	2.8	4.5	-1.0	7.5
Refraction, cylinder, right eye (D)	54		-1.5	1.0	-1.3	-2.3	-0.5	-4.3	-0.3
Refraction, sphere, center eye (D)	54		3.3	1.7	3.1	2.3	4.3	-0.8	8.5
Refraction, cylinder, center eye (D)	54		-1.4	0.9	-1.3	-1.8	-0.8	-4.3	0.0
Sphere difference between the 2 eyes (D)	54		0.9	0.7	0.7	0.5	1.3	0.0	2.7
Cylinder difference between the 2 eyes (D)	54		0.8	0.7	0.7	0.3	1.0	0.0	3.0
Incomplete retinal vascularization	29/53	54.7							
Papillary excavation	0/53	0							
ROP at TEA	5/53	9.4							
Pathological MRI	16/54	31.5							
Subtentorial or fossa posterior lesions	7/54	13.0							
Sustentorial lesions	15/54	27.8							
VSA	54		7285	595	7263	6958	7612	5908	8642
CSI	54		1.0	0.0	1.0	1.0	1.0	0.9	1.0


### Visual Acuity

Visual acuity was higher in premature infants at TEA than in the reference with term newborns at birth (1.54 ± 0.67 vs. 0.99 ± 0.40 cycles/degree, *p* = 0.008). No relationship was found between visual acuity and actual age or corrected age in VPI. Potential associated factors with higher visual acuity in bivariate analyses and included in the multivariate analysis are presented Table 3. Of note, the refraction of the two eyes in combined analysis did not reveal an impact of the sphere, but it did reveal a significant impact of the cylinder (right eye *r* = 0.031, left eye *r* = 0.219; *p* = 0.004).

**Table 3:** Visual acuity (cycles/degree) by risk factors in bivariate analysis.

Variables	Yes	No	*r*	*p*
Intraventricular hemorrhage	1.2 ± 0.6	1.6 ± 0.7		0.150
Early onset infection	0.9 ± 0.4	1.6 ± 0.7		0.060
Maternal consumption of alcohol during pregnancy	0.8 ± 0.0	1.6 ± 0.7		0.111
Pathological MRI	1.3 ± 0.3	1.6 ± 0.7		0.065
Subtentorial or fossa posterior lesions	1.1 ± 0.5	1.6 ± 0.7		0.157
Weight at the time of evaluation			0.0003	0.139
Higher cerebral VSA			0.003	0.068
Smaller CSI			-15.061	0.176


*r, regression coefficient in simple linear regression; p, p-value*

There was no significant association with the other factors studied (*p* > 0.2).

In multivariate analysis, abnormal brain MRI was the only factor associated with visual acuity (*r*^2^= 0.203, *p* = 0.026).

### Other Visual Development Parameters

No infant had signs of visual impairment. Among the observed palpebral abnormalities, 6 infants had bilateral port wine stains and 1 had a left eyelid hidrocystoma. No other morphological abnormalities were found. Eye pursuit was normal in all patients. FE revealed vascular tortuosity in three cases. No infant had papillary excavation, with a cup/disk ratio ≤0.2 in all cases.

Retinal vascularization at TEA was completed in only 24 of the premature infants. Incomplete retinal vascularization was associated with MRI abnormalities of the posterior fossa (*p* = 0.027) and a larger refractive sphere difference between both eyes (1.2 ± 0.8 vs. 0.6 ± 0.4 diopters; *p* = 0.0005). The Spearman correlation test did not demonstrate a correlation between head circumference at birth and the refractive measurements, or between head circumference at TEA and refractive measurements. However, a correlation of *r* = 0.306 (*p* = 0.024) was measured between head circumference at birth and at TEA, and a correlation of *r* = 0.275 (*p* = 0.044) between head circumference at birth and the refractive sphere difference between both eyes at TEA. Palpebral abnormalities were not significantly related to the refractive data. The blink reflex to the threat appeared to be associated with an absence of ROP at TEA (*p* = 0.042). We did not find any significant correlation between the glare reflex and environmental factors. The convergence reflex was related to the actual age at the time of the assessment; it was present at an average age of 99.1 ± 18 days and absent at an average age of 88 ± 13.7 days (*p* = 0.049). Regarding postconceptional age, it was present at an average age of 43.4 ± 2.3 weeks GA and absent at an average age of 42.1 ± 1.4 weeks GA (*p* = 0.076). No significant relationship was found with environmental factors.

### Retinopathy of Prematurity (ROP)

Thirteen of our patients had ROP in the neonatal period; they were born more premature (27.8 ± 1.6 vs. 30.0 ± 1.5 weeks GA, *p* = 0.0001) and with a lower birth weight (1002 ± 315 vs. 1248 ± 256 g; *p* = 0.007). In addition, they had longer duration of mechanical ventilation (11.4 ± 10.8 vs. 4.1 ± 5.4 days; *p* = 0.002), more moderate and severe BPD (80% vs. 29.4% and 20% vs. 5.9%, respectively; *p* = 0.001), more surgery for PDA (30.8% vs. 2.6%, *p* = 0.011), and more late onset infections (53.8% vs. 20.5%, *p* = 0.034). The maximum ROP stage, with both eyes combined, was stage 1 in 7.7% of infants, stage 2 in 61.5% of infants, and stage 3 in 30.8% of infants. The most severe zone reached with both eyes combined was zone II in 69.2% of cases and zone III in 30.8% of cases. For the five cases of ROP observed at TEA, the maximum stage was stage 1 in 1 infant and stage 2 in 4 infants. The most severe zone was zone II in 1 infant and zone III in 4 infants. Asymmetry of the ROP between both eyes was noted. No ROP treatment was necessary in our population. ROP in VPI was significantly associated with indicators of smaller cerebral volume (VSA = 6981.1 ± 606.2 vs. 7387.7 ± 579.0; *p* = 0.035).

## Discussion

Visual acuity was higher in premature infants at TEA than in term newborns at birth as reported by the Vital-Durand study (Vital-Durand, 1992). These studies are relevant for a comparison with our population because of the comparability of the measurement of visual acuity by the preferential looking method (Vital-Durand et al., 1996). This result is consistent with Searle et al. (1989) who showed that visual acuity of preterm infants born ≤ 36 weeks GA was better than full-term infants born > 36 weeks GA. Moreover, visual acuity development in healthy preterm infants appears to be accelerated when compared with full-term infants of the same post-conceptional age (Searle et al., 1989). This result may be related to a longer exogenous visual experience from birth in the VPI, to the contrary of the study of Weinacht et al. (1999) who showed that additional visual experience of preterm infants born after 31 weeks GA did not influence development of visual acuity during the 1st months of life.

Searle et al. (1989) showed that acuity development of normal preterm infants was related to post-conceptional age rather than actual age. Accordingly, we did not find an effect of the actual age at TEA on visual parameters in our population. A critical period for the onset of visual maturation is suggested in VPI. The neurological pathways of the retina are active between 22 and 40 weeks GA and may begin to function before the organization of sleep, which appears at approximately 28–30 weeks GA. Their organization occurs during sleep in the dark (Graven, 2004). However, total permanent darkness is deleterious for visual development; visual experience in the 1st months of life is essential for the development of visual function (Maurer and Lewis, 2001). Newborns exposed to a day and night luminous rhythm open their eyes more than those exposed to continuous light stimulation (Robinson et al., 1989). The entry of light into the eye is likely not limited in premature infants before 32 weeks PMA, as the eyelids are thin and often open (Graven, 2004). In our unit, we cover the incubators with a cloth in order to reduce the ambient brightness, especially direct exposure of the retina to strong light, which is known to be toxic (Niessen, 2006). In addition, the ambient lights are softened at night to facilitate sleep/wake rhythms. Thus, when the eyelids are closed, the children may have better darkness, which could lead to better organization of the retinal neurological pathways. The eyelids are closed for 55% of the time among those under 26 weeks GA, 93% of the time at 28 weeks GA, and 60% of the time at 34 weeks GA (Robinson et al., 1989; Fielder and Moseley, 2000). Therefore, the beginning of the critical period of visual maturation is likely around 28 weeks postmenstrual age (PMA). The number of patients is insufficient to show this developmental threshold effect at an intermediate age between 24 and 32 weeks, but this point should be taken into consideration in subsequent studies.

We only found an association between a lower visual acuity and abnormal cerebral MRI, without a significant specific link with the occipital area. This may be due to the small size of our population and a lack of power. Padilla et al reported significant global reductions in the brain tissue of premature infants born before 27 weeks GA but observed specific increases in cortical gray and white matter involving vision-related brain areas (Padilla et al., 2015).

Cerebral imaging is interesting for assessing the development of vision (Chau et al., 2013; Padilla et al., 2015; Naud et al., 2017). The cerebral volume seems related to visual development and the occurrence of abnormalities. Cerebral volume indices such as VSA, which is a standardized spherical surface area calculation (Naud et al., 2017), was significantly associated with visual acuity in the multivariate analysis. Given the interest of the VSA and the link between the development of the posterior fossa and the retina, it would be helpful to develop an indicator of occipital brain volume development. Beyond the usefulness of the VSA in physiological visual maturation, this indicator seems to provide meaningful information in pathological situations, as it is significantly lower with ROP. Sveinsdóttir et al. (2018) showed a relation between ROP and lower cerebellar volume and lower unmyelinated white matter volume at TEA. Moreover, Drost et al. (2018) showed an association between severe ROP and reduced cerebellar and brainstem volumes measured by automatic segmentation method at TEA. Both of those studies concluded to neurodevelopmental deficits at 2 years (Drost et al., 2018; Sveinsdóttir et al., 2018). For medical teams that do not have these measurement techniques available, VSA is an easy to measure indicator in routine MRI. We were not able to evaluate the measurements of optical radiation, particularly because of the poor quality of the diffusion images. It would be interesting to evaluate the occipital area specifically, particularly by fractional anisotropy measurements (Chau et al., 2013) or functional brain imaging (Padilla et al., 2015).

None of our patients showed signs of visual impairment at TEA. Although strabismus is more frequent in VPI (Denis et al., 2006) the absence of strabismus in our population at TEA may be due to the too young actual age of our infants. Strabismus is an abnormality of binocular vision, which is set up between 3 and 5 months of life (Denis et al., 2006; Niessen, 2006); thus, being able to diagnose it prior to that age is unlikely.

The convergence reflex usually appears after 4 months. The timing of the appearance of visual reflexes in VPI is not documented in the literature. Our results suggest that visual reflexes appear according to the actual age and not the corrected age because the convergence reflex tends to appear when approaching 4 months of actual age and not corrected age. Weinacht et al. (1999) suggested that convergence reflex depends on postconceptional age in low preterm infants. However, this difference can be explained by the difference of population as we studied more immature infants and because only 8 infants of our cohort had a convergence reflex at TEA.

Angiogenesis of the retina and brain visual pathway seems to play an important role in visual maturation. Of note, 54.7% of our VPI’s retinal vascularization were surprisingly yet not completed at TEA. In our study, incomplete vascularization was associated with larger refractive sphere difference. So, it is possible that the vascularization has a role on the development of the eyeballs and thus on the refractive parameters of the eyes. Retinal vascularization is never strictly symmetrical, as in the asymmetry of ROP. Hellgren et al. (2016) showed more anomalies of spherical equivalents at 30 months corrected age when infants had ROP with an effect of the severity of ROP. We did not highlight this element, perhaps because of the low prevalence of ROP in our cohort. The impact of retinal angiogenesis on volume development in the eyes of VPI would be interesting to evaluate. The incomplete retinal vascularization of VPI at TEA seems to be predictive of anisometropia.

Contrary to the results of Good and Hou (2015), we did not find a link between visual maturation at TEA and jaundice. This can be explained by the fact that none of our jaundice patients exceeded the neurotoxic threshold and were all treated with phototherapy.

The visual acuity at TEA was not different when ROP was present in the neonatal period. Because the macula is the precise vision area containing the largest number of cones and located in zone I, and none of the ROP in our population was in this zone, our finding seems to be consistent with previous data. Nevertheless, it would be interesting to evaluate the visual field at a later age.

Our study has limitations. First, the number of patients was rather small. Even though we found a number of significant associations, the non-significant trends we observed may be due to a lack of statistical power. Second, cycloplegia was performed using tropicamide due to contraindications at this age and the toxicity of other cycloplegic drugs in the context of prematurity. Tropicamide may be a less reliable cycloplegic drug and does not allow the establishment of optical correction at the age of our population. Whether the refraction performed at TEA by tropicamide can be predictive of abnormalities confirmed by a more reliable cycloplegic drug in follow-up would be interesting to assess, as it would allow greater vigilance and implementation of earlier treatment for the prevention of amblyopia.

## Conclusion

The critical period of onset of visual maturation of VPI remains to be defined. Evaluating the continuation of visual maturation in these VPI in order to obtain reference values for this population is important, as well as following the evolution of the visual parameters and defining any predictive elements of future amblyopia in order to improve care. A follow-up of our cohort is planned at 18 months corrected age, 4 and 7 years.

## Author Contributions

MW participated in the concept and design of the study, acquisition, analysis and interpretation of data, wrote and revised the manuscript. AN and ES participated in the acquisition of data and revised the manuscript. IC-U participated in analysis and interpretation of data. J-MH participated in the concept and design of the study, interpretation of data and revised the manuscript. All authors approved the manuscript as submitted.

## Conflict of Interest Statement

The authors declare that the research was conducted in the absence of any commercial or financial relationships that could be construed as a potential conflict of interest.

## Abbreviations

BPD: bronchopulmonary dysplasia
CSI: Cortex Skull Index
FE: fundus examination
GA: week’s gestation
MRI: magnetic resonance imaging
PDA: patent ductus arteriosus
ROP: retinopathy of prematurity
SD: standard deviation
TEA: term equivalent age
VPI: very preterm infants
VSA: Volume Slice Area
